# Healthcare professional acceptance of telemonitoring for chronic care patients in primary care

**DOI:** 10.1186/1472-6947-12-139

**Published:** 2012-11-30

**Authors:** José Asua, Estibalitz Orruño, Eva Reviriego, Marie Pierre Gagnon

**Affiliations:** 1 Department of Health and Consumer Affairs, Basque Government, Office for Health Technology Assessment (Osteba), Vitoria-Gasteiz, Spain; 2 Department of Health and Consumer Affairs, Basque Government, Direction of Knowledge Management and Evaluation, Vitoria-Gasteiz, Spain; 3 Faculty of Nursing Sciences, Université Laval, Québec, Canada; 4 Research Centre of the Centre Hospitalier Universitaire de Québec, Québec, Canada

**Keywords:** Telemonitoring, Chronic care patients, Healthcare professional, Technology Acceptance Model (TAM), Primary care, Basque country, Psychosocial factors

## Abstract

**Background:**

A pilot experimentation of a telemonitoring system for chronic care patients is conducted in the Bilbao Primary Care Health Region (Basque Country, Spain). It seems important to understand the factors related to healthcare professionals’ acceptance of this new technology in order to inform its extension to the whole healthcare system.

This study aims to examine the psychosocial factors related to telemonitoring acceptance among healthcare professionals and to apply a theory-based instrument.

**Methods:**

A validated questionnaire, based on an extension of the Technology Acceptance Model (TAM), was distributed to a total of 605 nurses, general practitioners and paediatricians. Logistic regression analysis was performed to test the theoretical model. Adjusted odds ratios (OR) and their 95% confidence intervals (CI) were computed.

**Results:**

A response rate of 44.3% was achieved. The original TAM model was good at predicting intention to use the telemonitoring system. However, the extended model, that included other theoretical variables, was more powerful. Perceived Usefulness, Compatibility, and Facilitators were the significant predictors of intention. A detailed analysis showed that intention to use telemonitoring was best predicted by healthcare professionals’ beliefs that they would obtain adequate training and technical support and that telemonitoring would require important changes in their practice.

**Conclusion:**

The extended TAM explained a significant portion of the variance in healthcare professionals' intention to use a telemonitoring system for chronic care patients in primary care. The perception of facilitators in the organisational context is the most important variable to consider for increasing healthcare professionals’ intention to use the new technology.

## Background

Chronic diseases are a leading health concern worldwide and the Basque Country (Spain) has recently adopted a whitepaper in order to foster an integrated chronic care strategy [1]. Telemonitoring systems are increasingly implemented for the follow-up of chronic diseases because they allow capturing patients’ clinical parameters (*e.g.* heart rate, blood pressure, blood oxygen saturation, blood glucose, electrocardiograph, respiratory flow, etc.) easily and in a continuous or intermittent pace [2]. Telemonitoring also enhances self-management where patients are often in charge of transferring self-measured clinical data through the system either to primary care professionals or to a specialised care centre where the received parameters can be integrated with other relevant information related to the state of the patient [2].

A systematic review showed that data transmitted through telemonitoring systems demonstrated a high level of accuracy and reliability [3]. Furthermore, the processes of data transfer of various telemonitoring systems has proven to be effective, and limited technical problems and errors were detected [3]. With respect to patients’ attitudes and behaviours, telemonitoring technologies have generally been well received and accepted [3]. Studies also report that home telemonitoring is cost-effective for the follow-up of high-risk pregnant women [4], patients suffering from heart failure [5] and those with chronic obstructive pulmonary disease (COPD) [6].

Telemonitoring has been found to reduce rates of hospitalisation and emergency department visits for COPD patients [7-9]. Telemonitoring of diabetic patients is associated with a significant improvement in glycemic control [10,11]. In the case of asthma, telemonitoring has shown significant improvements in patients’ peak expiratory flows, considerable reductions in symptoms related to the disease, and improvements in perceived quality of life [10].

With respect to telemonitoring interventions for patients with heart failure, previous studies report their effectiveness in reducing the risk of all-cause mortality, heart failure-related hospitalisations, emergency department visits, as well as improving patient self-care, perceived quality of life, evidence-based prescribing and overall control of the disease [8,12-16]. In the UK a recent evaluation of the Whole System Demonstrator project, a large pragmatic cluster-randomized trial of telemonitoring for chronic disease, shows decreased hospital admission and mortality among patients receiving the telemonitoring intervention [17]. However, recent large-scale trials of telemonitoring for patients with heart failure found no clinical benefit [18,19].

Patient acceptance of telemonitoring systems is essential in order to ensure the success of this new care modality [20]. However, healthcare professionals are also pivotal for the success of a telehealth program because this requires important changes in their usual practice [21].

While physician acceptance of telemedicine has been extensively studied [22-27], only a few studies have specifically explored healthcare providers’ acceptance of telemonitoring systems. In the United Kingdom, Sharma and colleagues [28] investigated clinicians’ perceptions toward telemonitoring of patients with chronic conditions using the concepts of Giddens’s Structuration Theory and Consequence of Modernity [29,30]. Their findings showed that trust and sense of security emerged as the two concepts that determined clinicians’ adoption of telemonitoring. A study conducted in Quebec (Canada) evaluated non-physician healthcare professionals' adoption of elder home care telemonitoring [31,32]. This study was based on Triandis’ Theory of Interpersonal Behavior [33] but employed a qualitative research design. Habits and perceived barriers in clinical practice were identified as the main determinants of healthcare professionals’ adoption of elder home care telemonitoring.

Our team has conducted a previous study on healthcare professionals’ adoption of a hospital-based telemonitoring system at the Donostia University Hospital (Gipuzkoa, Basque Country) [34]. Using a modified version of the Technology Acceptance Model (TAM) proposed by Davis [35], we found that facilitating conditions were the most important predictor of hospital physicians’ and nurses’ intention to use a telemonitoring system. This previous work allowed us to suggest modifications to the theoretical model and to develop an enhanced instrument that could better capture the factors influencing healthcare professionals’ acceptance of telemonitoring.

The aim of this study was twofold: 1) to evaluate the determinants of healthcare professionals’ acceptance of a telemonitoring system for the management of chronic patients in primary care; and 2) to apply an adapted theory-based instrument to assess healthcare professionals’ acceptance of telemonitoring.

## Methods

### Context of the study

The present study is part of a home telemonitoring clinical trial (the TELBIL study) conducted across 23 health centres of the Bilbao Primary Health Care Region, Basque Country (Spain) [36]. The health region serves a catchment population of 390,000 people, of whom around 27% are over 60 years old. The team of professionals in charge of the first level of healthcare is composed of 239 general practitioners (GPs), 326 nurses and 40 paediatricians. Between February 1^st^, 2010 and August 31^st^, 2011 a primary care-based randomized clinical trial (RCT) was carried out to evaluate the impact of a telemonitoring intervention aimed at home care patients with hearth failure and/or chronic lung disease (trial registration: ISRCTN89041993). In-home patients, diagnosed with heart failure and/or chronic lung disease, aged 14 or above and with two or more hospital admissions in the previous year were eligible for the trial. Fifty-eight patients were recruited for the study (28 in the intervention group and 30 in the control group).

In addition to the usual care, the intervention group was followed-up through telemonitoring, which consisted of daily transmissions of patient self-measurements of respiratory rate, heart rate, blood pressure, oxygen saturation, weight and body temperature. Additionally, patients in the intervention group completed a qualitative symptom questionnaire daily using the telemonitoring system. If the measurements received at the primary health centre fell outside the established limits, alerts were triggered via the PDA terminal and the clinical staff acted according to the medical condition of the patient. The control group received usual care, consisting of regular medical examinations in line with the established programs for the monitoring of home-based chronic patients.

The primary outcome measure of the RCT was the number of hospital admissions due to any cause that occurred in a period of 12 months post-randomization. Secondary outcome measures included: duration of hospital stay, hospital admissions due to heart failure or chronic lung disease, mortality rate, use of healthcare resources, quality of life, cost-effectiveness, compliance and patient and health professional satisfaction with the new technology. The results of this trial are reported elsewhere [37].

Our study was conducted during the first months of the trial but did not specifically target providers who took part in it. In fact, all of the 23 healthcare organisations agreed to participate, but patients were recruited in only a portion of them. Thus, the purpose of this study was to understand the factors that could influence healthcare providers’ acceptance of telemonitoring in order to inform implementation strategies for a future scaling-up of this service.

The study was approved by the Ethics Committee for Scientific Research (CEIC, Basurto Hospital, Bizkaia) on the 16^th^ of December 2009.

### Theoretical framework

Our theoretical framework is an extension of the Technology Acceptance Model (TAM) proposed by Davis [35]. The TAM was developed to understand user’s acceptance of information technology. In its original version, the TAM considers intention as the direct antecedent of behaviour, while Perceived Ease of Use (PEU) and Perceived Usefulness (PU) are regarded as the predictors of intention. However, several authors have questioned the applicability of the TAM to understand the behaviours of healthcare professionals [23,24]. Modifications and extensions have been proposed in order to increase the predictive value of the TAM [38,39] and to improve its applicability to the healthcare sector [22,24,27]. However no study to date has quantitatively applied a technology acceptance model to the specific context of telemonitoring for patients with chronic diseases in primary care.

**Figure 1:**
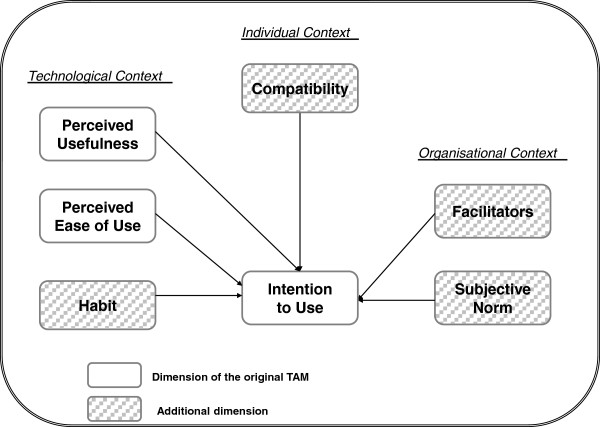
Adapted theoretical model.

Our proposed extended model is based mainly on Chau and Hu’s model of telemedicine acceptance [22] and comprises three dimensions: the individual context, the technological context and the organisational context. The individual context encompasses the variables Attitude, which represents the potential adopter’s perception of the positive or negative consequences related to adopting the technology, and Compatibility, which refers to the degree of correspondence between an innovation and existing values, past experiences and needs of the potential adopter [40]. The technological context includes the variables Perceived Ease of Use (PEU) and Perceived Usefulness (PU) as well as Habit. Habit was proposed by Triandis in his Theory of Interpersonal Behavior (TIB) and refers to behaviour that has become automatized [33]. With respect to the organisational context, the variables Subjective Norm and Facilitators are added in our theoretical model. Subjective Norm originates from the Theory of Reasoned Action [35,41] and assesses the extent to which the potential adopter believes that people who are important to him or her will approve his or her adopting the system. The variable Facilitators (or facilitating conditions) originates from the TIB [33] and refers to the degree to which the potential adopter believes that an organisational and technical infrastructure exists to support the use of the system. The adapted theoretical model is presented in Figure 1.

### Data collection

Data were collected by means of a questionnaire that was adapted from our previous work [23,34,42] to this particular study. The questionnaire was first developed based on items derived from an elicitation of salient beliefs regarding telehealth among physicians [23] and validated in a study among healthcare professionals (nurses and physicians) of a tertiary hospital in the Basque Country [34]. The internal consistency of the instrument was assessed by calculating the Cronbach alpha values for each theoretical variable. The construct validity of the model was evaluated using inter-item correlation analysis. With the exception of Habit, Cronbach alpha values were acceptably high (≥ 0.7) for the remaining theoretical constructs. Given the low internal consistency of the variable Habit, the questions addressing previous experience of use of telemonitoring and other ICT in clinical practice were included with the socio-demographic characteristics of participants. Thus, we considered previous experience as an external variable and verified its influence on intention. Furthermore, the measures of Attitude and PU showed multicollinearity in our previous study because these concepts are closely related, both measuring the perceived consequences of adopting a behaviour. Since Attitude is more generic than PU, the former was removed from our theoretical model in the present study.

The study questionnaire was in Spanish and comprised 36 questions (see Additional file 1 for the English version). The same questionnaire was used for nurses, GPs and paediatricians. A link to an electronic version of the questionnaire was sent by email to the professional electronic address of all nurses, GPs and paediatricians of the Bilbao Primary Health Care Region. One week after sending the questionnaire, a first reminder was sent to all participants by email. We were not able to target non-respondents only because the study was anonymous. This email first thanked participants who had already completed the online questionnaire and provided the link to the questionnaire for those who did not respond. A total of three reminder emails were sent (one after each week). Lastly, paper copies of the questionnaire were sent to each primary health care centre of the health region and a person was mandated to distribute them to nurses, GPs and paediatricians who had not completed the electronic survey.

Each theoretical item was assessed on a 7*-*point Likert scale, ranging from −3 'totally disagree' to +3 'totally agree'*.* To facilitate analysis and interpretation, we recoded the scores on a scale from 1 to 7. Scores were developed by computing the mean of all the items that form each theoretical dimension. Additionally, respondents had to provide information about their age, gender, medical specialty, number of years in clinical practice, highest educational grade obtained, and previous use of information technology. There were no particular incentives to participation.

### Statistical analysis

First, we conducted descriptive statistics to examine the mean and standard deviation of the scores of the theoretical variables. We also computed and examined the correlations between the theoretical variables. Since the dependent variable (Intention to Use) was not normally distributed, we performed a logistic regression analysis. The dependent variable was dichotomised by choosing the median (5.0) as the cut-off point, as recommended by psychosocial theorists [43]. The values for this variable were 'low to moderate intention = 0' and 'high intention = 1'. In other words, the respondents having a mean score for intention to use of 5 or higher were categorised as high intenders and those with a mean score for intention to use below 5 were categorised as low to moderate intenders. In total, 117 respondents were classified as 'high intender' and 151 as 'low to moderate intenders'.

Second, we explored the influence of participants’ socio-demographic characteristics (age, gender, medical specialty, number of years in clinical practice, highest grade obtained, and previous experience with telemonitoring and other ICT applications) on their intention level using non-parametric tests.

Third, we conducted a logistic regression with blocking of variables. The first block comprised the TAM variables, namely PU and PEU, followed by additional theoretical variables (Subjective Norm, Facilitators, Compatibility and Habit) in the second block. Fourth, following a method proposed by von Haeften and colleagues [44], we explored the contribution of individual items (or beliefs) underlying each of the significant theoretical constructs to predict the intention.

Finally, we developed a parsimonious logistic regression model predicting healthcare professionals’ intention to use telemonitoring based on the more important beliefs identified in the previous step. A statistical significance threshold of 0.05 was chosen. Adjusted odds ratios (OR) and their 95% confidence intervals (CI) were computed. The analyses were performed using SPSS software version 19 (SPSS Inc., Chicago IL).

## Results

### Internal consistency

Cronbach alpha values were acceptable (> 0.7) for the dependent variable (Intention to Use) and the variables PU, PEU, Subjective Norms and Facilitators (Table 1). By contrast, the Cronbach alpha value was slightly low for Compatibility (α = 0.61). An acceptable alpha value (α = 0.80) was obtained by eliminating the item COM4 (item no. 34) “The use of telemonitoring could interfere with the usual follow-up of my patients”. For the variable Habit, only the item “I have already used telemonitoring devices to monitor my patients” was used so no Cronbach alpha was calculated.

**Table 1:** Internal consistency of theoretical dimensions

**Dimension**	Items used^*^	**Sample item**	**Cronbach α**
Perceived usefulness	11, 15, 19, 23, 27 and 32	The use of the telemonitoring system (TMS) could help me to monitor my patients more rapidly	0.96
Perceived ease of use	12, 16, 20, 24, 28 and 35	I think that I could easily learn how to use the TMS	0.91
Compatibility	14, 21, 29 and 34	The use of the TMS may imply major changes in my clinical practice	0.80^†^
Subjective norm	17, 22, 26 and 31	Most of my patients will welcome the fact that I use the TMS	0.85
Facilitators	18, 30 and 36	I think that my health centre has the necessary infrastructure to support my use of the TMS	0.82
Habit^‡^	9	I have already used telemonitoring devices to monitor my patients	---
Intention	13, 25 and 33	I have the intention to use the TMS when it becomes available in my health centre	0.90

^*^ See Supplementary file for the complete questionnaire.

^†^ When the item 34 is dropped.

^‡^ The variable Habit was assessed using a single item. No Cronbach alpha was calculated.

### Characteristics of the participating healthcare professionals

Overall, 605 questionnaires were sent to all GPs (N = 239), paediatricians (N = 40) and nurses (N = 326) of the Bilbao Primary Health Care Region. A total of 268 professionals (122 GPs, 15 paediatricians, and 131 nurses) responded (see Table 2). All of the 23 health care centres of the region were represented, but participation within each organisation varied between 1 and 17 respondents. The overall response rate to the questionnaire was 44.3%. Almost 80% of respondents were women and the main age group represented (78.4%) was between 40 and 59 years old*.* Respondents had on average 21.3 years in clinical practice. Available administrative data only allowed us to compare our sample to the whole population on the variables professional category and age group. We performed chi-square tests and found no significant difference (data not shown).

**Table 2:** Participants’ characteristics

**Characteristics**	**All participants**		**Nurses**		**GPs**		**Paediatricians**	
	**(n = 268)**		**(n = 131)**		**(n = 122)**		**(n = 15)**	
	**n**	**%**	**n**	**%**	**n**	**%**	**n**	**%**
Gender								
Women	209	78.0	124	94.7	75	61.5	10	66.7
Men	59	22.0	7	5.3	47	38.5	5	33.3
Age								
<30	11	4.1	11	8.4	0	0	0	0
30-39	43	16.0	24	18.3	15	12.3	4	26.7
40-49	93	34.7	42	32.1	46	37.7	5	33.3
50-59	117	43.7	52	39.7	60	49.2	5	33.3
>60	4	1.5	2	1.5	1	0.8	1	6.7
Spoken language								
Castellano	250	93.3	125	95.4	113	92.6	12	80.0
Euskera	18	6.7	6	4.6	9	7.4	3	20.0
Years in clinical practice	21.3 (SD=9.1)		22.6 (SD=9.7)		20.2 (SD=8.3)		19.7 (SD=7.5)	
Highest educational grade								
3-year certificate	116	43.3	116	88.5	0	0	0	0
B.Sc.	114	42.5	6	4.6	99	81.1	9	60.0
M.Sc.	18	6.7	8	6.1	9	7.4	1	6.7
Ph.D.	10	3.7	0	0	6	4.9	4	26.7
Other studies	10	3.7	1	0.8	8	6.6	1	6.7

### Descriptive statistics of the theoretical variables

Table 3 reports the descriptive statistics of the theoretical variables. Healthcare professionals’ acceptance of the telemonitoring system was moderate, with a mean score for PU and PEU of 4.70 and 5.07, respectively. Respondents also had a positive perception of facilitating conditions, with a mean score of 5.08. Theoretical constructs were well correlated with each other and with the dependent variable (Intention to use) and no multicollinearity was detected.

**Table 3:** Descriptive analysis of the theoretical variables

	**PU**	**PEU**	**COM**	**SN**	**FAC**	**IU**
Mean	4.70	5.07	4.85	4.68	5.08	4.86
SD	1.40	1.13	1.25	1.10	1.22	1.32
Correlation with IU	0.83*	0.72*	0.81*	0.65*	0.83*	1.00*

PU= Perceived usefulness SN**=** Subjective Norm.

PEU= Perceived ease of use FAC**=**Facilitators.

COM=Compatibility IU=Intention to use.

* Correlation significant at p = 0.01.

### Influence of external variables on the intention

The number of high intenders and low intenders were compared between professional categories using the chi-square test. GPs and paediatricians were grouped given the small number of paediatricians. There was no significant difference between nurses and physicians (data not shown). The influence of other socio-demographic characteristics (gender, age group, number of years in clinical practice, highest grade obtained, and previous experience with telemonitoring and other ICT applications) on the level of intention was also explored. None of these relationships were significant (data not shown).

### Logistic regression

Table 4 presents the results of the logistic regression of the original TAM model (with PU and PEU as the sole predictors of Intention), and the extended model. Both models fitted the data well, as confirmed by the Hosmer and Lemeshow tests that were not significant. First, the core TAM model was tested comprising the variables PU and PEU. The chi-square was significant for this block and the Nagelkerke R^2^ was 0.63. That means that 63% of the variance in the Intention can be explained by the core TAM model. Both the variables PU and PEU are significant with odds ratios of 5.28 (95%CI: 3.14-10.01) and 1.93 (95%CI: 1.11-2.37), respectively.

**Table 4:** Results of the logistic regression: Original and Extended TAM

**Independent variables**	**Multivariate regression OR**	**95% CI**	**p**
**Original TAM**^**a**^			
Perceived usefulness	5.28	3.14-10.01	0.000
Perceived ease of use	1.93	1.11-2.37	0.020
**Extended TAM**^**b**^			
Perceived usefulness	2.65	1.15-6.12	0.022
Perceived ease of use	0.66	0.31-1.39	0.276
Compatibility	3.06	1.30-7.18	0.010
Subjective Norm	1.06	0.56-2.03	0.851
Facilitators	4.90	2.38-10.09	0.000
Habit	2.56	0.56-11.70	0.226

TAM=Technology Acceptance Model; OR: Odds Ratio; CI: confidence interval.

^**a**^ Nagelkerke R^2^ = 0.63 ^**b**^ Nagelkerke R^2^ = 0.72.

Second, the added theoretical variables (Social Norms, Facilitators, Compatibility and Habit) were entered in block into the regression. As shown in Table 4, the model is still significant and slightly more powerful (Nagelkerke R^2^ = 0.72). Seventy-two percent of the variance in Intention is thus explained by the extended model. Three variables significantly predict intention: Facilitators (OR = 4.90, 95%CI: 2.38-10.09), Compatibility (OR = 3.06, 95%CI: 1.30-7.18) and PU (OR = 2.65, 95%CI: 1.15-6.12). This means that the variable Facilitators accounts for most of the odds of having a high intention to use telemonitoring.

We explored the items or beliefs that had the most influence on healthcare professionals’ intention, following a method proposed by von Haeften [44]. First, all items forming the three significant predictors (Facilitators, Compatibility and PU) were entered in a logistic regression model. This led to the identification of the three most critical beliefs, two referring to Facilitators and one to Compatibility. As shown in Table 5, a final logistic regression was performed with these three items alone in order to test a more parsimonious and specific model explaining healthcare professionals’ intention to use telemonitoring. This model is slightly less powerful than the previous model (Nagelkerke R^2^ = 0.68), and the three critical items are, in order of importance: “I am willing to use the telemonitoring system if I receive the necessary technical assistance” (OR = 2.70, 95%CI: 1.44-5.05), “I am willing to use the telemonitoring system if I receive adequate training” (OR = 2.36, 95%CI: 1.20-4.62), and “Using the telemonitoring system may imply important changes in my clinical practice” (OR = 2.30, 95%CI: 1.65-3.22).

**Table 5:** Results of the logistic regression based on items from theoretical constructs

**Items from theoretical constructs**	**Multivariate regression OR**	**95% CI**	**p**
**FAC3:***I am willing to use the TMS if I receive the necessary technical assistance*	2.70	1.44-5.05	0.002
**FAC2:***I am willing to use the TMS if I receive adequate training*	2.36	1.20-4.62	0.013
**COM1:***Using the TMS may imply major changes in my clinical practice*	2.30	1.65-3.22	0.000

FAC=Facilitators; COM=Compability; OR: Odds Ratio; CI: confidence interval.

Nagelkerke R^2^ = 0.68.

## Discussion

This study shows that healthcare professionals in primary care have the intention to adopt telemonitoring for the management of chronic care patients. This finding is important because nurses and physicians are regarded as the most important gatekeepers for telehealth services [26]. Given that most patients are informed of telehealth programmes by their healthcare providers, they could, in turn, be more willing to receive telemonitoring services if they perceive support from nurses and physicians. In addition, patient decision of whether to use telemonitoring or not depends mainly on providers’ willingness to offer this service [26]. Thus, healthcare providers have a direct role to play in the implementation and diffusion of telemonitoring services.

The perception of facilitators to telemonitoring adoption, the compatibility of this technology with clinical practices and its perceived usefulness are the most influential variables in the prediction of nurses’, GPs’, and paediatricians’ intention to use this new technology. The results of this study are consistent with those of a previous study conducted by our team that assessed hospital clinicians’ intention to adopt a telemonitoring system [34]. These results are also similar to those reported in a study of healthcare professionals’ adoption of teledermatology [42]. Again, the perception of facilitators was identified as the most important factor in the prediction of healthcare professionals’ intention to use teledermatology.

Furthermore, this study identifies specific items that are likely to increase the odds of healthcare professionals to move from a low intention to a high intention to use telemonitoring system in their practice. For instance, the availability of training and adequate technical support is likely to foster providers’ acceptance of telemonitoring for the management of chronic care patients in primary care. These elements should, thus, be central in communication strategies directed at healthcare professionals in order to prepare them for a large scale implementation of the telemonitoring system experimented in this pilot trial. Another belief that is likely to impact on healthcare professionals’ intention to use the telemonitoring system is the fact that this technology would require important changes in their practice. Surprisingly, this belief is positively related to intention, meaning that healthcare professionals do not see the expected change negatively. In the literature, resistance to change has often been associated with poor adoption of information and communication technologies by healthcare professionals [45]. Our finding contrasts with previous studies, but it could indicate that the healthcare professionals who were surveyed have already prepared themselves for a change in their practice, and could be more ready to adopt the technology when largely available.

The results of this study partly support the proposed extended TAM framework, based largely on Chau & Hu’s model of telemedicine acceptance [22]. However, our model indicates that facilitators have the strongest influence on intention, thus eliminating the direct effects of PEU on intention. Habit was not found to influence intention significantly and this could be due to the lack of previous experience with telemonitoring among the study participants. The indirect effects of PU and PEU on intention through facilitators were not tested in the present study but represent important steps for further validation of the proposed model. Furthermore, our study validates the Spanish version of the instrument that we have developed to measure the psychosocial determinants of telemonitoring acceptance by healthcare providers and confirms that it could be applied in both hospital and primary care settings.

### Study limitations

The results of our study should be interpreted in light of some limitations. First, despite the fact that the response rate corresponds to that of other similar studies [34,42], it remains limited and participation bias cannot be excluded.

Furthermore, it was not possible to document differences between healthcare professionals who participated in this survey and those who did not because data on sociodemographic characteristics (gender, spoken language, experience) is not recorded in the health region administrative database. Consequently, it is not possible to assess the generalizability of our results to the whole population of healthcare providers of the health region. Nonetheless, we were able to show that the sample was not significantly different from the population with respect to professional category and age group.

Also, there could be a participation bias related to completing an online questionnaire. We tried to limit this potential bias by offering the possibility to non-respondents to complete a paper-based questionnaire in order to reach the healthcare professionals who do not use their electronic mail. However, only a small number of professionals completed the paper-based version of the questionnaire and we could not compare them with those who completed the online questionnaire.

Another limitation of this study is that the measures of the psychosocial constructs are based on items from the literature and from a questionnaire developed in our previous studies [23,34,42]. Theoreticians from social psychology suggest that these variables should be based on the salient beliefs of the specific population under study [46]. However, a set of predefined beliefs is usually employed to assess the theoretical constructs of the TAM [35]. In our study, we have tried to adapt the phrasing of the items to increase their relevance to the studied behaviour and the particular context, but some important beliefs regarding telemonitoring adoption in the target population might have been overlooked.

Our theoretical model encompasses several constructs belonging to psychosocial theories that have proven to be effective in explaining the behaviours and intentions of healthcare professionals [47]. However, other variables could also be important in explaining healthcare professionals’ intentions to use telemedicine applications, such as professional and personal norms [23]. Other technology acceptance models have been tested among healthcare providers and it would be important to assess the relative contribution of the proposed determinants. Using more advanced statistical methods such as structural equation modelling is highly recommended but data should respect the conditions regarding normality, homoscedasticity, independence and linearity. It also requires and large numbers of participants, which is not always feasible in real life implementation studies.

Finally, we did not assess actual use of telemonitoring because a limited number of respondents had access to the telemonitoring service due to the limited number of devices available in this pilot. However, the literature shows that intention is a good proxy for actual behaviour among healthcare professionals [48].

## Conclusion

In response to the increasing burden of chronic diseases, healthcare systems look for alternative solutions in order to improve clinical outcomes and limit costs. Consequently, telemonitoring is increasingly seen as an efficient and cost-effective solution. Telemonitoring is associated with improved health outcomes and increased patient involvement in all aspects of their own care. Healthcare providers’ acceptance of this new system represents one of the key issues that must be addressed in order to ensure the successful implementation of telemonitoring programs. This study supports the validity of the extended Technology Acceptance Model in order to explain the main barriers and facilitators to healthcare professionals’ adoption of a telemonitoring system. This study confirms that the most important factors that influence healthcare professionals’ acceptance of telemonitoring are related to the organisational context and comprise appropriate training and support. Telemonitoring acceptance is also related to the perceived compatibility of this technology with the changing practice of primary healthcare providers related chronic care management. Strategies to support a large-scale implementation of telemonitoring for chronic care patients in primary care should focus on healthcare professionals’ perceptions of being supported by their organisation and reinforced in their changing practices.

## Competing interests

The authors declare that they have no competing interests.

## Authors’ contributions

JA obtained funding, contributed to the questionnaire development and validation, and revised the manuscript. EO elaborated the questionnaire, supervised the electronic and paper distribution of the questionnaires, conducted data analysis, and collaborated to the manuscript redaction. MPG proposed the theoretical framework, contributed to the questionnaire development, collaborated to data analysis and wrote the first draft of the manuscript. ER prepared the Web version of the questionnaire, conducted the electronic distribution of the questionnaire and carried out the data collection. All authors reviewed and approved the final version of the manuscript.

## Pre-publication history

The pre-publication history for this paper can be accessed here:

http://www.biomedcentral.com/1472-6947/12/139/prepub

## Supplementary Material

Additional file 1 What’s your opinion on home telemonitoring? Click here for file
