# Supplementary material for: Healthcare professional acceptance of telemonitoring for chronic care patients in primary care
Source: BMC Med Inform Decis Mak. 2012 Nov 30;12:139. doi: 10.1186/1472-6947-12-139 (PMC3520721; doi:10.1186/1472-6947-12-139)
Supplement: Additional file 1 — What’s your opinion on home telemonitoring? [file 1472-6947-12-139-S1.pdf]

¿What's your opinion on home telemonitoring ?

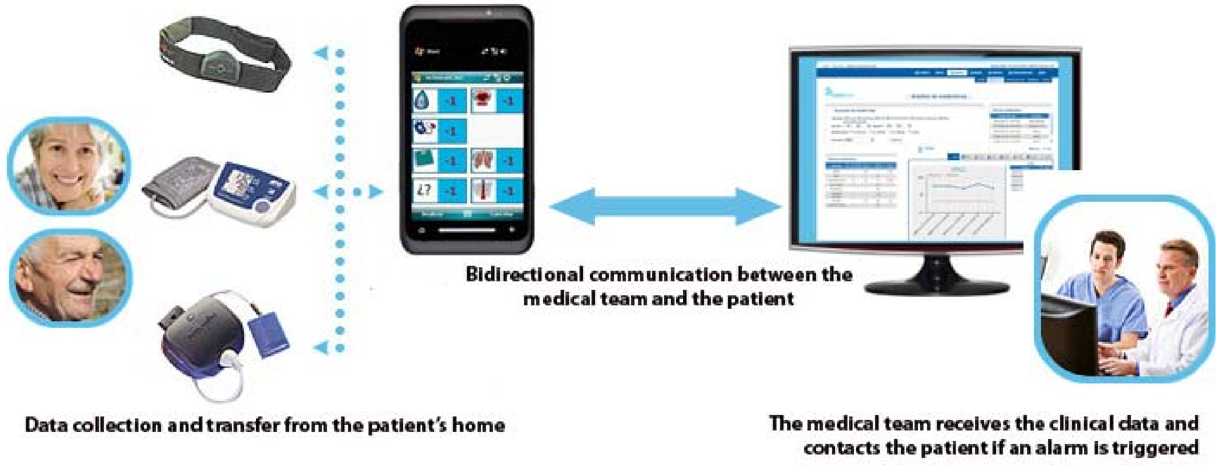

PURPOSE OF THE QUESTIONNAIRE

To find out the opinion of health care professionals from Bilbao Primary Care Health Region on home telemonitoring.

The objective of this study is to find out your opinion on this new technology. Having had previous experience with home telemonitoring is not necessary to answer the questionnaire. The information will be analyzed confidentially.

Please, complete this questionnaire. Your opinion is important.

1. Sex:

☐ Male ☐ Female

2. Age:

☐ < 30 years
☐ 30-39 years
☐ 40-49 years
☐ 50-59 years
☐ > 60 years

3. Medical specialty:

☐ General physician
☐ Nurse
☐ Paediatrician

4. Your health centre : ____

5. Number of years in clinical practice:____________________________________

6. Highest degree obtained (you can choose more than one option):

☐ 3-year certificate
☐ B.Sc.
☐ M.Sc.
☐ Ph.D.
☐ Other studies (Please specify):

____

7. I often use computing tools in my work:

☐ Yes
☐ No

8. I feel comfortable with information and communication technologies (e.g. e-mail, Internet, videoconference, on-line teaching, etc.):

☐ Yes
☐ No

9. I have already used telemonitoring devices to monitor my patients:

☐ Yes
☐ No

10. I am going to participate in the TELBIL study.

☐ Yes
☐ No

Here are 26 statements related to various factors that may be involved in the acceptance of telemonitoring as a working tool. Please, indicate your level of agreement with each of the following statements using the scale provided below:

** Remember to **select a single option** for each statement.

**-3**<br>**Totally disagree**	**-2**<br>Disagree	**-1**<br>Slightly disagree	**0**<br>Neither agree nor disagree	**1**<br>Slightly agree	**2**<br>Agree	**3**<br>**Totally agree**

	-3	-2	-1	0	1	2	3
11. The use of the telemonitoring system (TMS) could help me to monitor my patients more rapidly	☐	☐	☐	☐	☐	☐	☐
12. I think that I could easily learn how to use the TMS	☐	☐	☐	☐	☐	☐	☐
13. I have the intention to use the TMS when it becomes available in my health centre	☐	☐	☐	☐	☐	☐	☐
14. The use of the TMS may imply major changes in my clinical practice	☐	☐	☐	☐	☐	☐	☐
15. The use of the TMS could improve the monitoring of my patients	☐	☐	☐	☐	☐	☐	☐
16. I think that it would be easy to perform the tasks necessary for the monitoring of my patients using the TMS	☐	☐	☐	☐	☐	☐	☐
17. Most of my patients will welcome the fact that I use the TMS	☐	☐	☐	☐	☐	☐	☐
18. I think that my health centre has the necessary infrastructure to support my use of the TMS	☐	☐	☐	☐	☐	☐	☐
19. Using the TMS could help me get the most out of my time to monitor my patients	☐	☐	☐	☐	☐	☐	☐
20. I believe that the monitoring carried out by TMS would be clear and easy to understand	☐	☐	☐	☐	☐	☐	☐
21. The use of the TMS is compatible with my work habits	☐	☐	☐	☐	☐	☐	☐
22. Most of my colleagues will welcome the fact that I use the TMS	☐	☐	☐	☐	☐	☐	☐
23. Using the TMS could improve my performance in patients care	☐	☐	☐	☐	☐	☐	☐

-3 Totally disagree	-2 Disagree	-1 Slightly disagree	0 Neither agree nor disagree	1 Slightly agree	2 Agree	3 Totally agree

	-3	-2	-1	0	1	2	3
24. I think that the TMS is a flexible technology to interact with	☐	☐	☐	☐	☐	☐	☐
25. I have the intention to use the TMS when necessary to provide health care to my patients	☐	☐	☐	☐	☐	☐	☐
26. Health managers would welcome the fact that I use the TMS	☐	☐	☐	☐	☐	☐	☐
27. Using the TMS could facilitate the care of my patients	☐	☐	☐	☐	☐	☐	☐
28. I think I would find it easy to acquire the necessary skills to use the TMS	☐	☐	☐	☐	☐	☐	☐
29. The use of the TMS could promote good clinical practice	☐	☐	☐	☐	☐	☐	☐
30. I would use the TMS if I receive appropriate training	☐	☐	☐	☐	☐	☐	☐
31. Other health professionals (specialist, nurses, GPs etc.) would welcome the fact that I use the TMS	☐	☐	☐	☐	☐	☐	☐
32. In general, the TMS could be useful to improve the care of my patients	☐	☐	☐	☐	☐	☐	☐
33. I have the intention to use the TMS routinely for the care of my patients	☐	☐	☐	☐	☐	☐	☐
34. The use of the TMS could interfere with the usual follow-up of my patients	☐	☐	☐	☐	☐	☐	☐
35. I think that the TMS will be easy to use	☐	☐	☐	☐	☐	☐	☐
36. I would use the TMS if I receive the necessary technical assistance	☐	☐	☐	☐	☐	☐	☐

Do you have any comments?

____

____

Thank you very much for your cooperation!
